# Metabolism of Carotenoids and β-Ionone Are Mediated by Carotenogenic Genes and *PpCCD4* Under Ultraviolet B Irradiation and During Fruit Ripening

**DOI:** 10.3389/fpls.2022.814677

**Published:** 2022-05-13

**Authors:** Hongru Liu, Xiangmei Cao, Muhammad Azam, Chunfang Wang, Chenxia Liu, Yongjin Qiao, Bo Zhang

**Affiliations:** ^1^Crop Breeding & Cultivation Research Institute, Shanghai Academy of Agricultural Sciences, Shanghai, China; ^2^Research Center for Agricultural Products Preservation and Processing, Shanghai Academy of Agricultural Sciences, Shanghai, China; ^3^Laboratory of Fruit Quality Biology Zhejiang Provincial Key Laboratory of Horticultural Plant Integrative Biology, Zhejiang University, Hangzhou, China; ^4^Pomology Laboratory, Institute of Horticultural Sciences, University of Agriculture Faisalabad, Faisalabad, Pakistan

**Keywords:** carotenoids, β-ionone, metabolism, peach, UV-B irradiation

## Abstract

Carotenoids are essential pigments widely distributed in tissues and organs of higher plants, contributing to color, photosynthesis, photoprotection, nutrition, and flavor in plants. White- or yellow-fleshed colors in peach were determined by expression of carotenoids cleavage dioxygenase (*PpCCD*) genes, catalyzing the degradation of carotenoids. The cracked volatile apocarotenoids are the main contributors to peach aroma and flavor with low sensory threshold concentration. However, the detailed regulatory roles of carotenoids metabolism genes remained unclear under UV-B irradiation. In our study, metabolic balance between carotenoids and apocarotenoids was regulated by the expression of phytoene synthase (*PSY*), β-cyclase (*LCY-B*), ε*-*cyclase (*LCY-E*), and *PpCCD4* under UV-B irradiation. The transcript levels of *PpPSY*, *PpLCY-B*, *PpLCY-E*, and *PpCHY-B* were elevated 2- to 10-fold compared with control, corresponding to a nearly 30% increase of carotenoids content after 6 h UV-B irradiation. Interestingly, the total carotenoids content decreased by nearly 60% after 48 h of storage, while UV-B delayed the decline of lutein and β-carotene. The transcript level of *PpLCY-E* increased 17.83-fold compared to control, partially slowing the decline rate of lutein under UV-B irradiation. In addition, the transcript level of *PpCCD4* decreased to 30% of control after 48 h UV-B irradiation, in accordance with the dramatic reduction of apocarotenoid volatiles and the delayed decrease of β-carotene. Besides, β-ionone content was elevated by ethylene treatment, and accumulation dramatically accelerated at full ripeness. Taken together, UV-B radiation mediated the metabolic balance of carotenoid biosynthesis and catabolism by controlling the transcript levels of *PpPSY*, *PpLCY-B*, *PpLCY-E*, and *PpCCD4* in peach, and the transcript level of *PpCCD4* showed a positive relationship with the accumulation of β-ionone during the ripening process. However, the detailed catalytic activity of PpCCD4 with various carotenoid substrates needs to be studied further, and the key transcript factors involved in the regulation of metabolism between carotenoids and apocarotenoids need to be clarified.

## Introduction

Carotenoids are nutritional lipid-soluble tetraterpenoid pigments, which are widely distributed in a number of fruits, flowers, and vegetables, contributing to their colors, aromas, flavors, attractants for pollinators, and seed-spreaders (Nisar et al., 2015; Hou et al., 2016). Those compounds play important roles in light harvesting of photosynthesis system and photoprotection by scavenging free radicals and quenching single oxygen derived from excess light damage (Bouvier et al., 2005). In addition, carotenoids can be oxidatively cleaved by CCDs, producing norisoprenoid aroma volatiles, which are important contributors to floral aroma in fruits and flowers (Nisar et al., 2015). Their derivatives have been involved in a number of physiological processes in plants, including development and ripening of fruits, rhythmicity of flower volatile emission, metabolic of phytohormones, and small signaling molecules (Bouvier et al., 2005; Cazzonelli and Pogson, 2010; Nisar et al., 2015). Meanwhile, carotenoids have a beneficial effect on human health, being precursors of vitamin A and partial constituents, and have been used to reduce the risk of age-related macular degeneration and certain cancers (Kopsell and Kopsell, 2006; Yuan et al., 2015; Hou et al., 2016).

Carotenoids are composed of 40-carbon hydrocarbons derived from the building blocks of isopentenyl diphosphate (IPP) and dimethylallyl diphosphate (DMAPP). The basic five-carbon intermediates are produced by methylerythritol 4-phosphate (MEP) and mevalonic acid (MVA) pathways in the plastid and cytoplasm, respectively, in plants (Farré et al., 2010). Then geranylgeranyl pyrophosphate (GGPP), a common substrate of carotenoids and diterpenes, is condensed with three IPPs and one DMAPP molecule by geranylgeranyl pyrophosphate synthase (GGPPS) in a plastid. Then rate-limiting enzyme PSY converts two GGPP molecules to the first carotenoid phytoene, guiding carbon flux to the biosynthesis of carotenoids (Cazzonelli and Pogson, 2010; Fu et al., 2014). Phytoene is then transformed to lycopene by continuous desaturation and isomerization with enzyme catalyzation (Nisar et al., 2015). Subsequently, lycopene is cyclized with bifurcate pathway, either by alone *LCY-B*/chromoplast-specific lycopene β-cyclase (CYCB) to form β-carotene or by *LCY-B* and *LCY-E* to synthesize α-carotene (Alquezar et al., 2009). Moreover, α-carotene and β-carotene will be converted to lutein and zeaxanthin, respectively, *via* a hydroxylation reaction. Furthermore, the xanthophyll cycle between zeaxanthin and violaxanthin has been known to respond to the excess light stress (De Poll et al., 2011). Epoxidation of zeaxanthin is catalyzed by zeaxanthin epoxidase (ZEP) and reversed by violaxanthin de-epoxidase (VDE) (Chen and Gallie, 2012). Violaxanthin can be converted into neoxanthin by neoxanthin synthase (NSY), and 9-*cis*-violaxanthin can be cleaved at 11, 12 site and/or 9′-*cis*-neoxanthin at 11′, and 12′ double bond by 9-*cis*-epoxycarotenoid dioxygenase (NCED) to produce the precursor of abscisic acid (ABA), xanthoxin (Walter et al., 2010; Nisar et al., 2015). The catabolic of ABA is mainly catalyzed by the key enzyme of ABA8′-hydroxylase (Vallabhaneni and Wurtzel, 2010). Therefore, several rate-limiting enzymes determined the carbon flux of carotenoids metabolism, and the relationship between key enzymes and carotenoids constituencies has been intensively studied in plants (Nisar et al., 2015).

Carotenoids content are determined by the balance of biosynthesis and catabolism; unlike biosynthesis, which is determined by several key enzymes, the catabolism is mainly determined by CCD enzymes (Ibdah et al., 2006; Huang et al., 2009; Havaux, 2014; Hou et al., 2016). Various products have been produced by the oxidization of carotenoids with different cleaved sites, such as norisoprenoid volatiles, phytohormones (ABA, SLs), and some other small molecular metabolites (Havaux, 2014; Hou et al., 2016). CCD gene families have commonly been divided into CCD and NCED subclasses. Generally, CCDs consist of four members (1, 4, 7, 8), and NCED contains five members (2, 3, 5, 6, 9) in *Arabidopsis* (Gonzalez-Jorge et al., 2013; Song et al., 2016). The five NCEDs were involved in the metabolism of ABA, and VP14 from maize was the first identified member of the family (Tan et al., 2003; Messing et al., 2010). *AtCCD7/MAX3* and *AtCCD8/MAX4* members are continuously involved in the biosynthesis of strigolactone in *Arabidopsis* (Booker et al., 2004; Walter et al., 2010). Meanwhile, various norisoprenoid volatiles have been produced by CCD1 enzyme in tomato, grape, petunia, rose damascena, and *Osmanthus fragrans Lour* (Simkin et al., 2004a,b; Mathieu et al., 2005; Huang et al., 2009; Baldermann et al., 2010).

In addition, the members of CCD4 have been reported to degrade carotenoids, resulting in formation of white color (Brandi et al., 2011; Yoshioka et al., 2012; Falchi et al., 2013; Ma et al., 2013a). White-fleshed potato tubers and peach fruit showed higher expression levels of *CCD4* than the yellow-fleshed mutants, and accumulation of carotenoids was decreased in white-fleshed varieties with C-_13_ norisoprenoid volatiles content was increased (Campbell et al., 2010; Brandi et al., 2011; Falchi et al., 2013; Ma et al., 2013b; Bai et al., 2016). Besides, many studies have mentioned that protein CCD4 catalyzed carotenoids into apocarotenoid volatiles among many plants’ tissues and organs. Two types of *CsCCD4* genes with different subcellular localization in crocus are involved in both the degradation of β-carotene and the release of β-ionone in stigma (Rubio-Moraga et al., 2014). BnaC3.CCD4 might use δ-and/or α-carotene as substrates to produce α-ionone and determine the white- or yellow-color of petals from *Brassica napus* (Zhang et al., 2015). The β-ionone was one of the most important flavor compounds in green and black tea, and CsCCD4 from *Camellia sinensis* could cleave β-carotene at 9,10 (9′,10′) sites to produce β-ionone during the withering process (Wang et al., 2020). Besides, β-cyclocitral, β-ionone, β-damascenone, and β-citraurin in plants, algae, fungi, and bacteria could be produced by enzymatic cleavage of CCDs, peroxidases (PODs) and lipoxygenases (LOXs) and even with non-enzymatic oxidation with various carotenoids substrates (Ma et al., 2013a; Bruno et al., 2016; Liang et al., 2021; Zheng et al., 2021).

Carotenoids and their derivatives have exhibited key roles in diverse metabolic processes during plant development, fruit ripening, and interaction with various environmental stimuli (Bouvier et al., 2005; Walter and Strack, 2011). Carotenoid metabolites and xanthophyll cycles have been involved in the response to microclimate light and UV-B attenuation in the field during grapeberry development (Joubert et al., 2016). The cleaved metabolites, such as β-ionone and β-cyclocitral, have been reported to be important signaling molecules involved in interaction between plants and insects (Booker et al., 2004; Wei et al., 2011; Havaux, 2014; Hou et al., 2016). Plants exposed to UV-B (280–315 nm) have been widely reported to induce the accumulation of phenolic compounds in plants for their antioxidant properties (De Poll et al., 2011; Gil et al., 2012; Zeng et al., 2020). Meanwhile, carotenoids and their derivatives have also been involved in protecting against UV radiation as signal molecules, though the concentration of those compounds is usually low in plant tissues (Ramel et al., 2013; Havaux, 2014).

Previous studies have reported the metabolic mechanisms of carotenoids and apocarotenoids and identified many genes and enzymes to illustrate the molecular mechanism (Liang et al., 2021). However, the detailed regulatory roles during the development, fruit ripening, and response to environment stress were remained unclear, especially for horticultural crops (Shi et al., 2020; Koschmieder et al., 2021). Our study reported the metabolic of carotenoids and apocarotenoids were regulated by transcription changes of *PpPSY*, *PpLCY-B*, *PpLCY-E*, and *PpCCD4* under UV-B irradiation in peach. Meanwhile, the correlation between *PpCCD4* and C_13_-norisoprenoid volatile β-ionone was analyzed during peach ripening. We clarified the key gene roles in carotenoids and apocarotenoids metabolic molecular mechanisms in peach, while the detailed enzyme activities of PpCCD4 with various carotenoid substrates, such as β-carotene, lutein, violaxanthin, and zeaxanthin, need to be detected further. The identification of transcription factors and illumination of transcriptional regulation mechanisms under the development, ripening, and interaction with environmental stimuli need to be strengthened.

## Materials and Methods

### Plant Materials and Treatments

Peach (*Prunus persica* L. Batsch cv. Hujingmilu) was obtained from a well-managed orchard in Fengxian district of Shanghai, China. The fruits were harvested at three ripening stages, 110 days after bloom (110 DAB), 113 DAB, 116 DAB, and stored at 20°C for 3 days after harvest. The fruits were transported to the laboratory immediately after harvest from the orchard. Healthy fruits with uniform size, no mechanical damage, pathogen-, and disease-free were selected for our experiments. Each treatment consists of three biological replicates with five fruits each. Peel was separated from flesh, frozen in liquid nitrogen, and stored at –80°C for further analysis. UV-B irradiation treatments were processed as our previous work (Liu et al., 2017), fruits with uniform maturity were selected and irradiated with a high intensity of 1.5 W m^–2^ and a low intensity of 0.4 W m^–2^ for different times at 20°C. Ethylene and 1-methylcycloproene (1-MCP) treatments were performed as in previous work (Wu et al., 2017, 2019). Briefly, the marketable mature fruits were selected to be used, and the fruits were sealed in a bucket with a fan embedded in the lid. The fruits were suffocated for 12 h with an ethylene concentration of 1 μl L^–1^, produced from the ethephon, and the competitive inhibitor 1-MCP treatment was performed with a concentration of 5 μl L^–1^, and the control fruits were sealed only with the same buckets.

### Determination of the Fruit Ripening Quality Index

The firmness of fruit was analyzed according to Zhang et al. (2010) using a texture analyzer (TA-XT2i Plus, Stable Micro system) fitted with a 7.9-mm diameter head. The penetration rate was 1 mm s^–1^ with a final depth of 10 mm, and the two opposite sides at the equator of the fruit were used to measure. Total soluble solids (TSS) were determined by an Atago PR-101α digital hang-held refractometer, as performed by Zhang et al. (2010). Briefly, two opposite slices at the equator of each fruit were used to evaluate TSS with three drops of juice per slice. Fruit color was measured with a reflectance spectrophotometer (Hunter Lab Mini Scan XE Plus colorimeter) at eight evenly distributed equatorial surface points of each fruit. The raw data values of L* (lightness, from black to white), a* (the degree of green-red variation), and b* (the variation between blue and yellow) were recorded, and the color index of red grapes (CIRG) was calculated using the formula CIRG = (180-H)/(L* + C), and H = arctan (b*/a*) and C = [(a*)^2^ + (b*)^2^]^0.5^.

### Aroma Volatiles Extraction and Determination by Head Space Gas Chromatography Mass Spectrometry

Aroma volatiles of peach peel were extracted and analyzed by head space gas chromatography mass spectrometry (HS-GC-MS) as our previous study (Wei et al., 2021). Frozen peel was ground into powder under liquid nitrogen, and 1 g of powder was immediately transferred into vials. To adjust the instrument, 30 μl of 2-Octanol (0.8 mg/ml) was added into the vials as internal standard. A solid-phase micro-extraction (SPME) needle containing a fiber coated with 65 μm of polydimethylsiloxane and divinylbenzene (PDMS-DVB) (Supeclo Co., Bellefonte, PA, United States) was used for volatile concentration. The determination was automatic injection with a SPME autosampler (Combi PAL, CTC Analytics, Agilent Technologies), connected to a 7890N gas chromatograph and a 5975C mass spectrometer from Agilent company. Volatiles were isolated with a DB-WAX capillary column (30 m × 0.25 mm i.d. × 0.25 μm film thickness; J&W Scientific, Folsom, CA, United States). Helium was used as a carrier gas with a 1.0 ml/min flow rate. The temperature program in oven started at 40°C and was increased to 100°C with a rate of 3°C/min and then to 245°C with 5°C/min. The column effluent was ionized by electron ionization (EI) at an energy of 70 eV with a transfer temperature of 250°C and a source temperature of 230°C. Volatiles were identified by comparing their electron ionization mass spectra with the NIST Mass Spectral Library (NIST-08) and the retention time of authentic standards. Quantification of volatiles was performed using the peak area of the internal standard as a reference based on total ion chromatogram.

### Isolation and Determination of Carotenoids by High Performance Liquid Chromatography (HPLC) Analysis

The extraction and determination of the carotenoids in peach peel was performed as previous reports (Zhou et al., 2007; Fu et al., 2014). Briefly, frozen peel was grounded finely under liquid nitrogen, and 100 mg of powder was used to extract with chloroform/methanol/50 mM Tris-buffer, pH 7.5. The chloroform phase was collected by 10 min of centrifugation at 10,000 × *g*, and the aqueous phases were re-extracted with chloroform. The two combined chloroform phases were finally dried under nitrogen gas. The residue was first dissolved in 20 μl of diethyl ether and then in 200 μl of 6% (W/V) KOH in methanol. After mixing fully, the tube was transferred into a heating unit for incubation at 60°C for 30 min in darkness. Tris-buffer was then added into the incubated mixture, and re-extraction was carried out as described above, and finally dried with nitrogen gas. Three replicates were used for the determination of each sample. HPLC analysis was performed using Waters Alliance 2695 system (Waters Corporation, United States) consisting of a 2695 module and a 2996 PDA detector, equipped with a reverse phase 5 μm (250 mm × 4.6 mm) C_30_ column and a 20 mm × 4.6 mm i.d. C_30_ guard (YMC Inc., Wilmington, NC, United States). Chromatography was carried out at 25°C with the elution program as previously described by Zhou et al. (2007). Carotenoids were identified on the basis of the same retention times and same spectral characteristics as standards, and peak areas were recorded at 450 nm. The quantification was performed according to the calibration curve of the corresponding standards.

### Transcript Level Analysis

Total RNA extraction protocol was performed as our previous study (Liu et al., 2017), and the high-quality RNA was used to prepare the libraries for high-throughput RNA-seq as the protocols described previously (Zhang et al., 2016). RNA purity and concentration were detected with NanoDrop 2000 (Thermo Fisher Scientific, Wilmington, DE, United States). RNA integrity was measured using the RNA Nano 6000 Assay kit on the Agilent Bioanalyzer 2100 system (Agilent Technologies, CA, United States). A total of 1 μg of RNA from five fruits per sample was used for the RNA sample preparations. Sequencing libraries were made with the NEBNext Ultra™ RNA Library Prep Kit for Illumina (NEB, United States) according to the manufacturer’s protocol. The RNA-seq raw data presented in the study can be found in the National Center for Biotechnology Information (NCBI) Short Red Archive database. The accession numbers PRJNA576753 and PRJNA57477 are for peach samples at different ripening processes and under ethylene and 1-MCP treatments, respectively, and the data have been published in a previous study (Cao et al., 2021), and SRP103523 represents the UV-B treatment, and the data have been published in a previous study (Liu et al., 2017).

### Statistics

Figures were produced using ORIGIN 8.0 (Microcal Software, Inc., Northampton, MA, United States). The two-sample significance test was calculated using unpaired Student’s *t-*test, and Duncan’s test of one-way analysis of variance (ANOVA) was used to test the significance level of multiple groups (*p* < 0.05) (SPSS 21.0; SPSS Inc., Chicago, IL, United States).

## Results

### Accumulation of Norisoprenoid Volatiles and Transcription of *PpCCD4* During Peach Ripening

**FIGURE 1:**
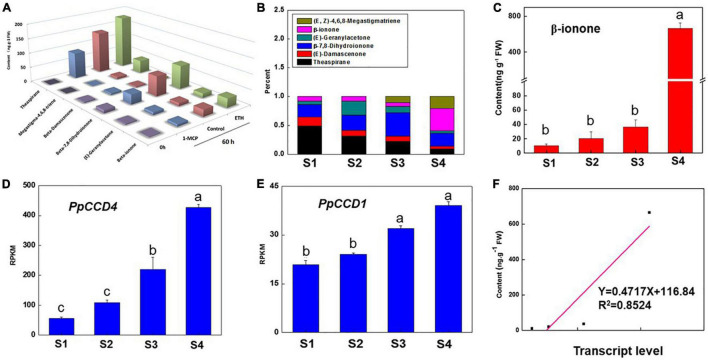
Change of the apocarotenoid volatiles and transcript level of *PpCCD4* during peach ripening. **(A)** Change of apocarotenoid volatiles under ethylene treatment. **(B)** Percentage change of the apocarotenoid volatiles under different ripening process, S1 represents 110 days after bloom (DAB), S2 represents 113 DAB, S3 represents 116 DAB, and S4 represents fruits that were stored at room temperature for 3 days. **(C)** Change of β-ionone content. **(D)** Transcript patterns of *PpCCD4*. **(E)** Transcript patterns of *PpCCD1*. **(F)** Correlation analysis between β-ionone content and transcript levels of *PpCCD4*. Different letters indicate the significant difference with Duncan’s multiple range tests at *p* < 0.05.

The peach ripening process is accelerated after the S1 color transition stage with the fruit quality index changing markedly. Fruit firmness decreased quickly from 44 N to 25 N in 1 week, and then decreased to 5 N within 3 days stored at room temperature after harvest (Supplementary Table 1). In accordance with ripening, the color index of CIRG and the fruit weight increased gradually, while the soluble solids content changed slightly except at the S1 stage (Supplementary Table 1). In addition, six norisoprenoid volatiles were detected throughout fruit ripening and under ethylene treatment (Figures 1A,B). Norisoprenoid volatiles content and proportions changed obviously as peach ripening and β-ionone proportion reached nearly 40% after 3 days storage at 20°C (Figure 1B). β-ionone content increased from S1 to S3 gradually, while the accumulation accelerated after S4 and reached nearly 670 ng g^–1^ FW with a 20-fold increase compared to S3 (Figure 1C). The proportion of theaspirane gradually declined from 48 to 8.7% in contrast with β-ionone increase during peach ripening (Figure 1B). Interestingly, (*E, Z*)-4,6,8-Megastigmatriene was detected at S3, and the content was increased at S4 stage (Figure 1B). In addition, the transcript level of *PpCCD4* showed an increasing trend as peach ripened, and was dramatically elevated at S4 (Figure 1D). *PpCCD4* expression was positively correlated with content change of β-ionone (*R*^2^ = 0.85, *p* < *0.05*) (Figure 1F), while the transcript level of *PpCCD1* increased gradually with low change in transcript levels (Figure 1E).

### Ethylene and 1-MCP Regulate β-Ionone Volatiles Content and *PpCCD4* Transcription

**FIGURE 2:**
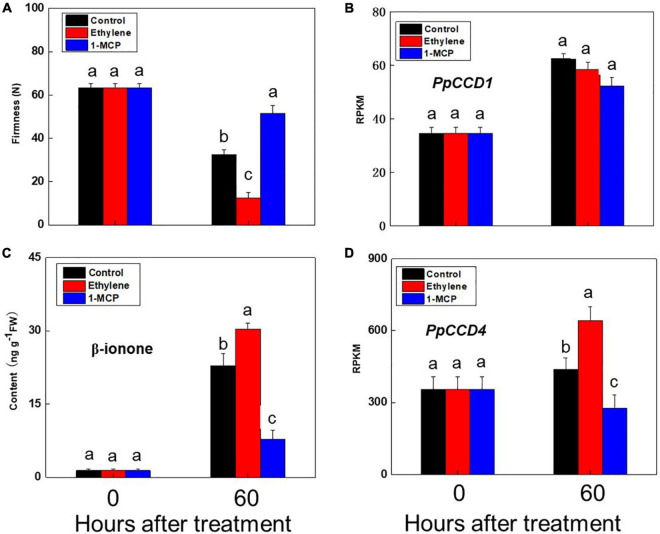
Change of β-ionone content and *PpCCDs* transcript levels after ethylene and 1-MCP treatments. **(A)** Change of the firmness. **(B)** Transcript level of PpCCD1. **(C)** Content of β-ionone. **(D)** Transcript level of *PpCCD4*. The fruits were suffocated with 12 h and then stored at room temperature for 60 h. Different letters indicate the significant difference between the two columns (*p* < 0.05).

Peach fruit exhibited climacteric characteristics as ripening, and ethylene played an important role in activating the ripening process. Accumulation of norisoprenoid volatiles and expression pattern of *PpCCD4* were evaluated under ethylene and 1-MCP treatments with fruits at the turning color stage (Figure 2). Ripening process of peach was significantly accelerated by ethylene suffocating, and firmness decreased significantly from 60 N to 10 N after 60 h of storage at room temperature (Figure 2A), while 1-MCP treatment markedly delayed the fruit ripening process with firmness changed slightly (Figure 2A). Accumulation of all the six kinds of norisoprenoid volatiles showed an increased trend after 60 h of storage, and ethylene treatment accumulated much higher content than control, while 1-MCP treatment inhibited accumulation of all six norisoprenoid volatiles (Figure 1A). The concentration of β-ionone was significantly higher than control under ethylene treatment after 60 h of room temperature storage, while accumulation was inhibited dramatically by 1-MCP treatment (Figure 2C). Meanwhile, transcript levels of *PpCCD1* showed no sensitivity to ethylene and 1-MCP treatments (Figure 2B). In summary, ethylene induced the accumulation of β-ionone by activating transcription of *PpCCD4*, while opposite effects were achieved by 1-MCP treatment.

### UV-B Irradiation-Mediated Carotenoids Metabolism in Peach

**FIGURE 3:**
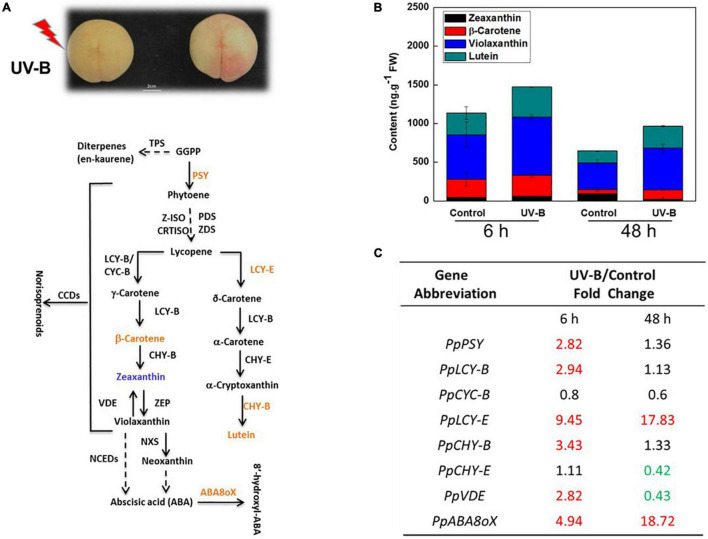
Carotenoids content and transcript level of genes regulated under UV-B irradiation. **(A)** The schematic map of carotenoids metabolic pathways. **(B)** Change of the carotenoids content. **(C)** Fold changes of the related genes transcript levels between UV-B and the control. The red color numbers mean the significant induction, and the green color mean the significant inhibition under UV-B irradiation with *p* < 0.05.

**FIGURE 4:**
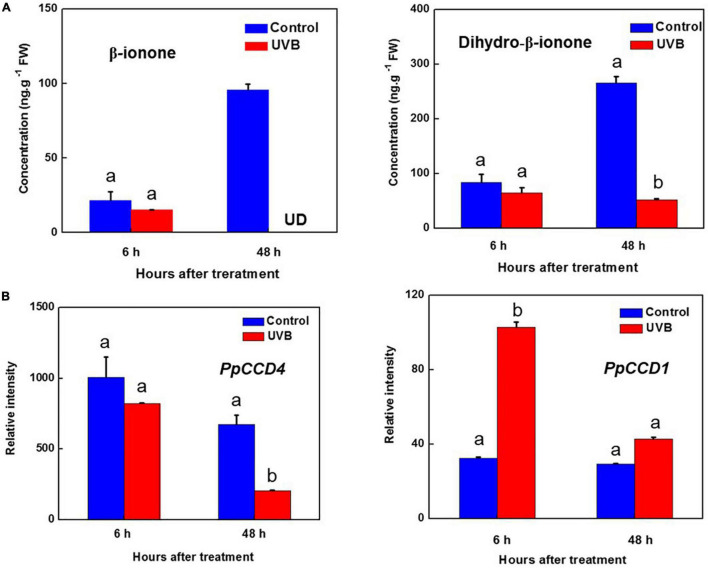
Transcript levels of *PpCCDs* and content of norisoprenoids changed by UV-B irradiation. **(A)** Content of the β-ionone and Dihydro-β-ionone. **(B)** Transcript levels of *PpCCD4* and *PpCCD1.* Different letters indicate significant differences between UV-B treatment and control (*p* < 0.05).

The color of the peach peel changed from pink to golden yellow after 48 h of UV-B irradiation (Figure 3A). The content of β-carotene, zeaxanthin, violaxanthin, and lutein was analyzed after 6 and 48 h of UV-B irradiation, respectively, in peach peel (Figure 3B). The lutein and violaxanthin accumulation occupied nearly 70% of the total four carotenoids, and β-carotene occupied 17% after 6 h of storage at room temperature (Figure 3B). Total content of the four carotenoids increased from 1,200 to 1,500 ng g^–1^ FW after 6 h of UV-B irradiation (Figure 3B). Numbers of key genes of carotenoids metabolism, such as *PSY*, *LCY-B*, *LCY-E*, and *CCDs*, have been identified in many higher plants (Fu et al., 2014; Nisar et al., 2015; Lado et al., 2016). Transcript levels of *PpPSY*, *PpLCY-B*, *PpLCY-E*, and *PpCHY-B* were significantly induced compared to control by 6 h of UV-B irradiation (Figure 3C and Supplementary Table 2). However, transcript levels of *PpPSY*, *PpLCY-B*, and *PpCHY-B* exhibited no significant difference compared with control except *PpLCY-E* after 48 h of UV-B irradiation (Figure 3C). After 48 h of storage at room temperature, total content of the four carotenoids reduced significantly compared with 6 h, indicating the biosynthesis of carotenoids was not the master factor in influencing carotenoids accumulation during fruit ripening (Figure 3B). Carotenoids proportion changed dramatically from 6 to 48 h in control group (Figure 3B). The proportion of β-carotene decreased from 21 to 9%, while the percentage of zeaxanthin increased from 4 to 14%, indicating the degradation of β-carotene was elevated during peach normal ripening (Figure 3B). However, the degradation of β-carotene was inhibited by UV-B irradiation, and β-carotene content accumulated 2-fold compared to control after 48 h of UV-B irradiation (Figure 3B). Meanwhile, the total content of downstream zeaxanthin and violaxanthin increased slightly by about 30% after 48 h of UV-B irradiation, prefiguring the other catabolism pathway catalyzed by CCDs that occupied the main proportion of β-carotene degradation and was inhibited after 48 h of UV-B irradiation (Figure 3A). In accordance with this, the transcript level of *PpCCD4* was significantly reduced after 48 h of UV-B irradiation (Figure 4B). On the contrary, the lutein accumulation increased significantly from 150 to 280 ng g^–1^, prefiguring that the biosynthesis of lutein contributed mostly to the accumulation at least after 48 h of UV-B irradiation for the induction of *PpLCY-E* transcript level (Figures 3B,C).

The biosynthesis and catabolism of carotenoids are determined by the numbers of genes labeled in the schematic map pathway, while usually several key genes in the pathway obviously influence the metabolic rates and directions (Figure 3A). The transcript level of the first rate-limiting gene, *PpPSY*, increased to 2.8-fold compared with control at 6 h of UV-B irradiation (Figure 3C). *PpLCY-B* controlled carbon metabolic flux to β-carotene access from lycopene, while *PpLCY-E* influenced the production of lutein (Figure 3A). Transcript levels of two vital branched genes *PpLCY-B*, and *PpLCY-E* were raised to 2.94- and 9.45-fold compared with control at 6 h of UV-B irradiation, respectively (Figure 3C). The downstream genes of β*-*β ring branched pathways *PpCHY-B, PpABA8oX* were all induced at 6 h of UV-B irradiation, indicating carotenoids catabolism and phytohormone ABA responded actively to UV-B (Figure 3C). However, transcript levels of the related genes were not induced as UV-B irradiation held for 48 h, except for *PpLCY-E* and *PpABA8oX* (Figure 3C). The continuous high transcript level of *PpLCY-E* supported the enhancement of lutein content after 48 h of UV-B irradiation (Figure 3B). *PpVDE* catalyzed violaxanthin to zeaxanthin and the transcript level changed from 2.82-fold induction to 57% inhibition during the UV-B irradiation duration, corresponding to a 78% reduction of zeaxanthin content after 48 h of UV-B irradiation (Figures 3B,C). Thus, carotenoids components and contents were influenced by transcript changes of different genes, and the metabolic sensitivity of carotenoids was increased by UV-B dose.

### *PpCCD4-*Mediated Change of β-Carotene and Norisoprenoid Volatiles Under UV-B Irradiation

Norisoprenoid volatiles were produced by cleavage of carotenoids, and β-carotene was one of the main substrates. Five kinds of norisoprenoid volatiles were detected in peach peel after 48 h of storage at room temperature (Supplementary Table 3). The content of dihydro-β-ionone and β-ionone increased significantly after 48 h of storage (Figure 4A and Supplementary Table 3). However, β-ionone content was nearly undetected after 48 h of UV-B irradiation, and its hydrogenation product of dihydro-β-ionone content decreased from 250 to 50 ng g^–1^ FW (Figure 4A). In common with content reduction of β-ionone and dihydro-β-ionone, the transcript level of *PpCCD4* decreased to 30% compared with control after 48 h of UV-B irradiation (Figure 4B). To further clarify the influence of UV-B, peach fruits were irradiated with different doses and intensities. The irradiation showed no obvious inhibition effects within 0.5 h, while, β-ionone content exhibited a significant reduction at 24 h of irradiation, and inhibition effects remained till 72 h of recovery in the dark after 24 h of UV-B irradiation (Supplementary Figure 1). The dose experiments showed that the inhibition effects were stronger under high intensity irradiation. Besides, the transcript level of *PpCCD1* was induced by UV-B irradiation at 6 h, indicating *PpCCD1* may play redundant roles in β-carotene catabolism and the formation of β-ionone (Figure 4B). Thus, UV-B irradiation decreased the biosynthesis of β-ionone by inhibiting the transcript level of *PpCCD4*.

## Discussion

Carotenoids are compositions of various color of plants organisms, such as a class of natural pigments that attract pollinators and dispersers for plants (Lewinsohn et al., 2005; Auldridge et al., 2006; Walter et al., 2010; Yuan et al., 2015). Carotenoids are also photosynthetic accessory pigments, participating in plant photosynthesis and photoprotection (Muller et al., 2001; Cerullo et al., 2002; Holt et al., 2005). In addition, carotenoids can be cleaved by CCD enzymes to produce apocarotenoids, some of which are key contributors to flavor and floral aroma in fruits and vegetables (Lewinsohn et al., 2005; Yuan et al., 2015). The structural diversity of apocarotenoids indicates the vital roles in plant–environment interactions (Walter et al., 2010; Havaux, 2014; Brousseau et al., 2021). The key enzymes of carotenoids biosynthesis and catabolism have been identified in many plants, such as loquat (Zhou et al., 2007; Fu et al., 2014), tomato (Simkin et al., 2004a; Lado et al., 2016), and citrus (Alquezar et al., 2009; Ma et al., 2013a; Rodrigo et al., 2013). Reports of PpCCD4 have mainly been limited to the genetic comparative analysis of white- and yellow-fleshed colors in peach varieties and carotenoids accumulation in leaves (Brandi et al., 2011; Falchi et al., 2013; Ma et al., 2013b). The molecular mechanism between carotenoids and volatile apocarotenoids still exists, especially under the abiotic stress. In our study, change of the main carotenoids and volatile apocarotenoids were determined and mediated by the key carotenogenic genes of the pathway and *PpCCD4* under UV-B irradiation. In addition, transcription of *PpCCD4* corresponded to the increase of β-ionone through peach ripening, in accordance with the ethylene and 1-MCP treatments.

### Change of Norisoprenoid Volatiles Were Correlated With Transcript Level of *PpCCD4* Through Peach Ripening and Ethylene Treatment

Norisoprenoid volatiles are widely distributed in fruits, flowers, and leaves of plants (Bouvier et al., 2005; Lewinsohn et al., 2005), exhibiting floral and fruity aroma attributes with low threshold concentration. Several reports have indicated that *PpCCD4* influenced carotenoids degradation and norisoprenoid volatiles accumulation in peach (Brandi et al., 2011; Falchi et al., 2013; Ma et al., 2013b). In our study, six main norisoprenoid volatiles were detected in peach, and their proportions changed significantly during the ripening process (Figures 1A,B). The proportion of theaspirane decreased gradually, while the proportion of β-ionone dramatically elevated at shelf-3-days, indicating β-ionone might contribute much to peach flavor aroma and seed dispersal (Figures 1B,C). Corresponding to this, the transcript level of *PpCCD4* was significantly induced as ripening (Figure 1D). The concentration of β-ionone and dihydro-β-ionone increased as the two apricot varieties ripened (Xi et al., 2020). To verify the metabolic mechanism of norisoprenoid volatiles through peach ripening, phytohormone ethylene and its competitive inhibitor 1-MCP were used to perform fumigation on turning color peach. Ethylene significantly promoted the peach ripening process with firmness decreasing markedly, while the fruit ripening process was obviously delayed under 1-MCP treatment with firmness decreasing less compared with the initial fruit (Figure 2A). Ethylene significantly elevated β-ionone content, while 1-MCP inhibited the accumulation of β-ionone dramatically, and transcript level of *PpCCD4* exhibited similar patterns (Figures 2C,D). All the six norisoprenoid volatiles were induced by ethylene and inhibited by 1-MCP treatments (Figure 1A). However, transcript level of *PpCCD1* showed no obvious sensitivity to ethylene and 1-MCP treatment (Figure 2B), which was corresponding to the change pattern of transcript level during peach ripening with no obvious response to ethylene peak at shelf-3 days (Figure 1E). Negative correlation was observed between levels of carotenoids and norisoprenoid volatiles *via* expression of *CCD1s* increased as fruit ripened in grapes (Mathieu et al., 2005), apricots (Xi et al., 2020), and watermelon (Lewinsohn et al., 2005). The positive correlation between *PpCCD4* transcript level and β-ionone content change indicated that *PpCCD4* was the potential biosynthetic gene of β-ionone in peach (Figure 1F), which was consistent with the previous reports (Brandi et al., 2011; Falchi et al., 2013; Bai et al., 2016).

### Carotenoids Metabolism Were Regulated by *PpPSY, PpLCY-E, PpLCY-B*, and *PpCCD4* Under UV-B Radiation

Carotenoids are mainly divided into two subgroups, namely, xanthophylls and carotenes, and some carotenoids are accessory pigments in photosynthesis by enhancing light harvesting, while some antioxidative xanthophylls can enhance plants’ tolerance to light damage by quenching reactive oxygen species induced by excessive irradiation (Muller et al., 2001; Cerullo et al., 2002; Robert et al., 2004; Holt et al., 2005). Carotenoids accumulation is associated with the expression of several key genes involved in the pathway, including *PSY*, *LCY-E*, *LCY-b*, *CHY-b*, and *VDE*, and is also regulated by related transcription factors, phytohormones, and abiotic stress (Marty et al., 2005; Welsch et al., 2007; Toledo-Ortiz et al., 2010). It is known that total carotenoids are commonly induced by UV irradiation. The field UV-B can alter β-carotene and lutein content during grapeberry development (Joubert et al., 2016). In our study, the transcript level of *PpPSY* has no significant increase after 48 h of UV-B irradiation, indicating that the total carbon flux flowing to carotenoids has no increase any more (Figure 3C). However, the transcript level of *PpLCY-E* continuously increased from 9.45- to 17.83-fold compared with control, resulting in the final product lutein that increased nearly 80% after 48 h of UV-B irradiation (Figures 3B,C). Lutein was involved in photoprotection of PSII system against the oxidative damage induced by excessed UV-B radiation in developmental grape berries (Biswas et al., 2020). Meanwhile, the production of lutein in wheat endosperm could be supplied amply with a very low expression of *LCYE-B* (Yu et al., 2021).

The content of β-carotene was regulated by expression of *PpPSY*, *PpLCY-B*, *PpCHY-B*, *PpVDE*, *PpCCD4*, and *PpCCD1* in β–βring branched pathway, and they were all induced at 6 h UV-B radiation except *PpCCD4* (Figures 3C, 4B). In accordance with the transcript level change, β-carotene content increased nearly 15%. However, the degradation of β-carotene by PpCCD4 played a vital role after 48 h of UV-B irradiation, for biosynthesis had no significant elevation while β-carotene content was markedly increased to 2-fold compared to control (Figure 3B). Corresponding to this, the transcript level of *PpCCD4* was significantly decreased to 30% compared to control after 48 h of UV-B irradiation (Figure 4B). Interestingly, the content of violaxanthin increased nearly 30% at 6 h of UV-B radiation, and the increase reached 56% compared to control after 48 h of UV-B irradiation (Figure 3B). Violaxanthin was deoxidized to zeaxanthin by *PpVDE*, and the deoxygenation process was significantly induced at 6 h with appropriate UV-B radiation, while the process was dramatically inhibited by 48 h UV-B radiation (Figure 3C). Accordingly, the content of zeaxanthin was significantly increased by 30% after 6 h of UV-B radiation, while the accumulation was markedly decreased by nearly 70% after 48 h of UV-B irradiation compared with control (Figure 3B). Violaxanthin has been known to have more resistance to high UV-B stress compared with other xanthophylls (Joshi et al., 2007; Lidon et al., 2012; Biswas et al., 2020), and the blocking of the violaxanthin de-epoxidation process by *PpVDE* indicated the photoprotection effect of zeaxanthin was limited under high UV-B irradiation. Two *PpNCEDs* (*PpNCED2, PpNCED3*) were surveyed in peach genome, and *PpNCED2* was induced slightly by UV-B, while *PpNCED3* had no response to UV-B irradiation (Supplementary Figure 2A). Besides, the transcript level of *PpNCED2* was elevated as the storage duration, which was corresponding to the induction pattern under the ethylene treatment (Supplementary Figure 2B). The transcript level of the *PpNCED2* was significantly increased from the turning color stage (S1) to the commercial maturity (Supplementary Figure 2C), which might be related to the metabolism of stress phytohormone ABA during fruit ripening.

### UV-B Controlled Formation of β-Ionone by Influencing Transcription of *PpCCD4*

UV-B influenced the metabolism of carotenoids by regulating key biosynthesis genes in the reaction chains. Meanwhile, catabolism of carotenoids, especially producing norisoprenoid volatiles by CCDs, was significantly influenced by UV-B irradiation, and the aroma flavor and colors of peach were changed. Transcript level of *PpCCD4* was decreased by high UV-B radiation at 48 h, while the influence was insignificant at 6 h of radiation (Figure 4B). In accordance with this, β-ionone content increased markedly in control, while the concentration was even under detected after 48 h of UV-B irradiation (Figure 4A). The content of dihydro-β-ionone occupied nearly 60% of the total norisoprenoid volatiles after 48 h of storage at room temperature (Supplementary Table 3), while UV-B irradiation decreased 80% of the dihydro-β-ionone content (Figure 4B). The inhibition of norisoprenoid volatiles content depended on UV-B irradiation intensity and doses, and the inhibition effect was obvious after 24 h of radiation and lasted till 72 h of recovery in the dark, and the high intensity (UVB-150) showed a stronger inhibition effect (Supplementary Figure 1). The norisoprenoid volatiles have been reported to be the substrates of glucosylation by the uridine diphosphate sugar-dependent glycosyltransferases (UGTs) in *N. benthamiana* and *M.* × *piperita* (Sun et al., 2020). The apocarotenoid modification reactions share partial key enzymes with the detoxification induced by xenobiotics and some reactive carbonyl species (Koschmieder et al., 2021). Thus, the apocarotenoids might have been modified further in peach fruit, and the non-volatile glycosylated dihydro-β-ionone has been detected in our previous reports (Wu et al., 2017, 2019). A total of 13 *PpUGTs* were induced after 48 h of UV-B irradiation, and the transcript levels of their products were positively correlated with the content change of glycosylated dihydro-β-ionone during development (Wu et al., 2017). Thus, the candidate *PpUGTs* may transfer the free β-ionone and dihydro-β-ionone to the non-volatile glycoslated form in peach (Supplementary Table 4). Transcript level of *PpCCD1* was significantly induced at 6 h of UV-B irradiation, while the induction was not continuous till 48 h of irradiation (Figure 4B). The study indicated that the transcript level of *PpCCD4* may determine the emission of norisoprenoid volatiles by catalyzing the carotenoids in peach, especially for β-carotene, and the process was significantly inhibited by UV-B irradiation. Interestingly, 15- and 60 min-exposure of freshly cut cantaloupe melon to UV radiation induced release of β-ionone, β-cyclocitral and geranylacetone for the inhibition effects on microbial growth (Lamikanra et al., 2002). Thus, in a more general context, carotenoids and their cleavage products participated in multiple facets of plant defense mechanisms and responded to various environmental stimuli.

## Conclusion

In our study, the metabolic and molecular mechanisms of carotenoids and apocarotenoids volatiles were clarified under UV-B irradiation and during the fruit ripening process. Concentration of carotenoids were elevated at 6 h of UV-B irradiation with the induction of *PpPSY*, *PpLCY-B, PpLCY-E*, and *PpCHY-B*, whereas accumulation pattern of carotenoids and apocarotenoid volatiles was mainly influenced by the transcript levels of *PpCCD4*, *PpLCYE*, and *PpVDE* after 48 h of UV-B irradiation. The inhibition of *PpCCD4* might cause the accumulation of β-carotene and the reduction of β-ionone and dihydro-β-ionone, and the content of lutein and violaxanthin might be caused by the induction of *PpLCYE* and inhibition of *PpVDE* after 48 h of UV-B irradiation. In addition, β-ionone content increased during peach ripening and was induced by ethylene treatment. In summary, peach can regulate constitutes of carotenoids and mediate metabolism balance between carotenoids and apocarotenoid volatiles in response to UV-B irradiation stress. Meanwhile, there are still some works needed to be studied further. Firstly, biochemical activities of the key enzymes need to be determined with wide carotenoids substrates in peach to help understand the detailed biological roles completely. Secondly, the downstream metabolism of lutein and β-ionone needs to be studied deeply and offer us more information about the metabolic mechanism of carotenoids and apocarotenoid volatiles under UV-B irradiation. Finally, the transcriptional regulatory mechanisms need to be studied further, and the related transcription factors need to be identified. Meanwhile, the ethylene and UV-B light signal transduction pathways offered us clues.

## Data Availability Statement

The original contributions presented in the study are included in the article/Supplementary Material, further inquiries can be directed to the corresponding authors.

## Author Contributions

BZ, HL, and YQ designed the experiments. HL and XC performed the experiments. HL, MA, CW, and CL analyzed the data and wrote the manuscript. All authors contributed the article and approved the submitted version.

## Conflict of Interest

The authors declare that the research was conducted in the absence of any commercial or financial relationships that could be construed as a potential conflict of interest.

## Publisher’s Note

All claims expressed in this article are solely those of the authors and do not necessarily represent those of their affiliated organizations, or those of the publisher, the editors and the reviewers. Any product that may be evaluated in this article, or claim that may be made by its manufacturer, is not guaranteed or endorsed by the publisher.
