# Supplementary material for: Metabolism of Carotenoids and β-Ionone Are Mediated by Carotenogenic Genes and PpCCD4 Under Ultraviolet B Irradiation and During Fruit Ripening
Source: Front Plant Sci. 2022 May 13;13:814677. doi: 10.3389/fpls.2022.814677 (PMC9136946; doi:10.3389/fpls.2022.814677)
Supplement: Supplementary file 1 [file Data_Sheet_1.pdf]

Title: **Metabolism of Carotenoids and β-ionone are Mediated by Carotenogenic Genes and *PpCCD4* under UV-B Irradiation and during Fruit Ripening**

Running title: UV-B Regulates Metabolism of Carotenoids

Supplementary Materials

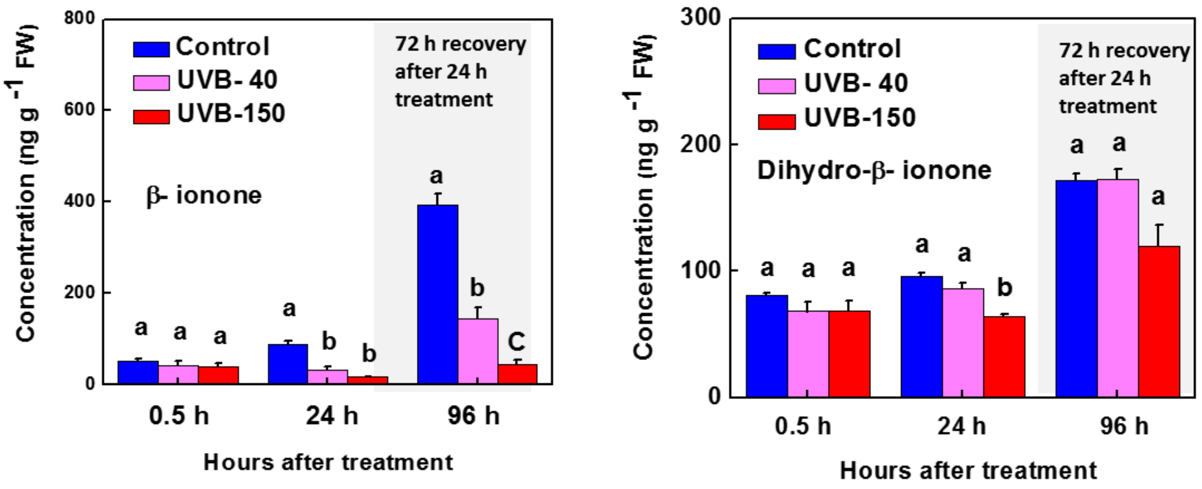

Supplementary Figure 1 | Change of β-ionone and Dihydro-β-ionone content under different dose of UV-B irradiation in peach.

UVB-40 means the intensity of 40 mw cm^{-2}, UVB-150 means the intensity of 150 mw cm^{-2}. Data are expressed as the means±standard error of three biological replicates. Significant differences are indicated with different letters for the same point differ significantly at $p < 0.05$ by Duncan's multiple range tests.

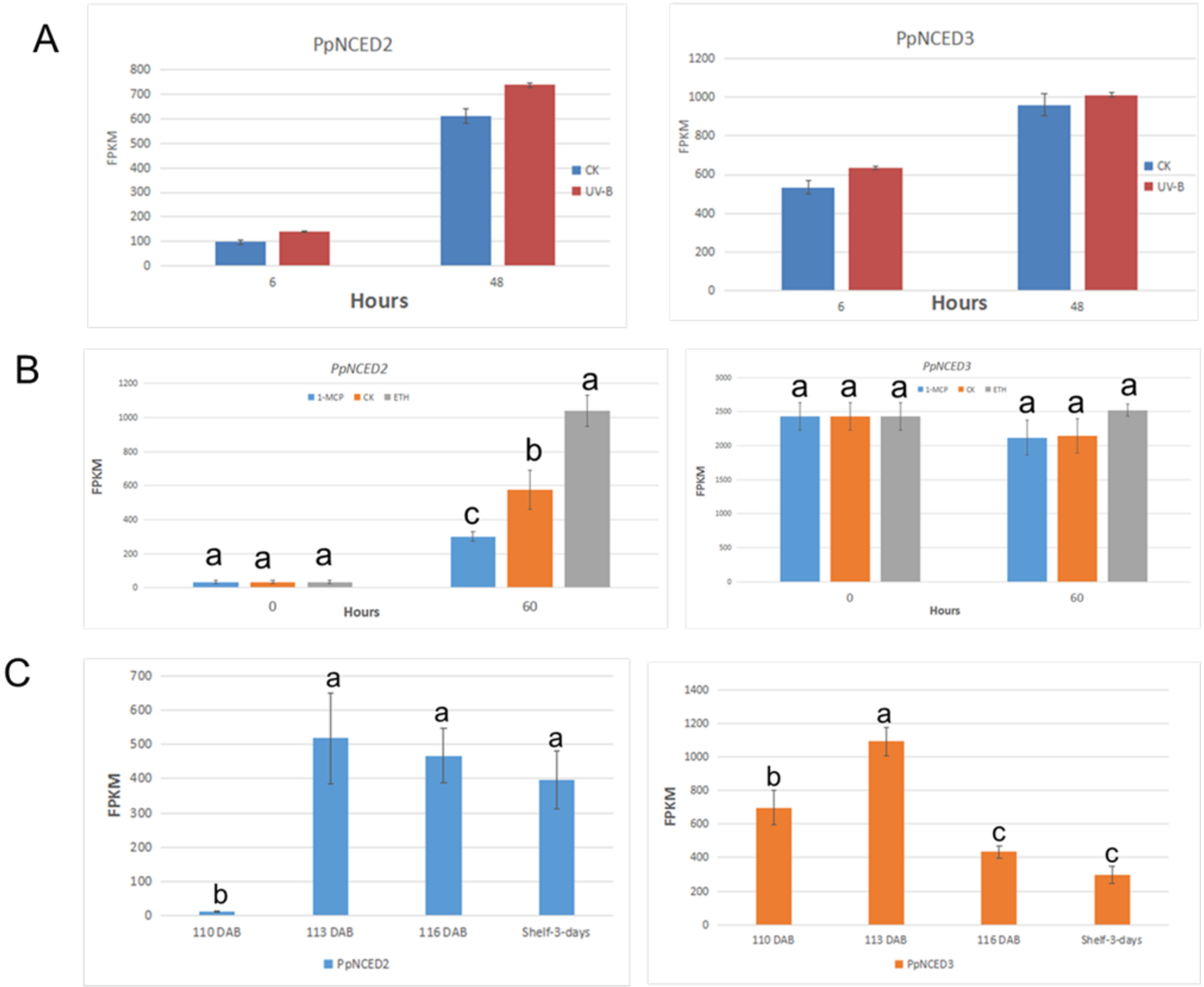

Supplementary Figure 2 | Transcript level of the two *PpNCEDs* under UV-B irradiation and during ripening process

Transcript level of *PpNCED2* and *PpNCED3* under UV-B irradiation; (B)Transcript level of *PpNCED2* and *PpNCED3* under ethylene and 1-MCP treatments; (C)Transcript level of *PpNCED2* and *PpNCED3* during ripening process. Significant differences are indicated with different letters at $p < 0.05$ by Duncan's multiple range tests.

Days after bloom	Fruit Weight (g)	TSS （brix）	Firmness(N)	CIRG
110DAB	160.34± 4.92 b	8.96± 0.18 b	44.55± 0.82 a	1.20± 0.05 c
113DAB	179.33± 7.70 b	10.09± 0.15 a	35.69± 1.04 b	1.53± 0.04 b
116DAB	211.99± 5.98 a	10.04±0.30 a	25.14± 2.31 c	1.75± 0.03 a
Shelf-3 Days	UD	9.77± 0.34 ab	4.64± 0.35 d	UD

Supplementary Table 1 | Physiological data during peach fruit ripening

Different letters of one column indicates the significant difference with Duncan's multiple range tests at $p<0.05$.

Abbreviation	GenBank accession
PpCHYB	Prupe.5G133900
PpCHYE	Prupe.7G123500
PpABA-OHB	Prupe.6G072400
PpLCY-B	Prupe.7G046100
PpLCY-E	Prupe.7G205600
PpVDE	Prupe.6G356100
PpABA-OHA	Prupe.5G013100
PpPSY	Prupe.3G178500
PpCCD4	Prupe.1G255500
PpCCD1	Prupe.2G014700

Supplementary Table 2 | Accession numbers of the genes used in the the paper

Name	6h CK	6h UV-B	48h CK	48h UV-B
Theaspirane	21.88±5.24	19.64±1.89	23.11±2.57	28.52±1.68
damascenone	98.27±15.83	73.30±5.59	52.89±11.27	31.15±4.23
β-7,8-Dihydroionone	79.67±13.24	68.29±9.04	258.58±13.23 a	50.45±1.56 b
Geranylacetone	32.16±9.38	16.07±3.06	24.55±5.13	23.56±1.49
β-ionone	22.78±4.34	14.26±0.37	92.69±3.24	Ud

Supplementary Table 3 | Norisoprenoid volatiles content of peach under UV-B radiation. Different letters indicate the significant difference of the two lines. Content units was (ng/g FW).

ID	UVB/CK
**Prupe.8G186200**	49.84
**Prupe.2G184800**	1.29
**Prupe.3G184400**	10.71
**Prupe.3G256000**	60.38
**Prupe.5G116100**	7.27
**Prupe.5G178200**	1.42
**Prupe.6G008400**	5.27
**Prupe.6G189900**	4.64
**Prupe.6G228400**	43.30
**Prupe.7G267900**	3.25
**Prupe.8G063500**	1.97
**Prupe.8G129800**	2.31
**Prupe.8G150700**	20.31

Supplementary Table 4 | Transcript level comparison of thirteen *PpUGTs* between UV-B and the control.
